# A case of metachronous peripheral T‐Cell non‐Hodgkin lymphoma following chemotherapy for Hodgkin disease successfully treated with brentuximab vedotin

**DOI:** 10.1002/ccr3.2898

**Published:** 2020-05-05

**Authors:** Federico Meconi, Ida Provenzano, Daniela Nasso, Benedetta Mariotti, Livio Pupo, Roberto Secchi, Raffaella Cerretti, Anemona Lucia, William Arcese, Maria Cantonetti

**Affiliations:** ^1^ Department of Biomedicine and Prevention University Tor Vergata Rome Italy; ^2^ Anatomic Pathology Department of Biomedicine and Prevention University of Rome Tor Vergata Rome Italy

**Keywords:** brentuximab vedotin, Hodgkin lymphoma, non‐Hodgkin T‐cell lymphoma

## Abstract

Occasionally, non‐Hodgkin lymphomas (NHL) occur simultaneously or subsequently to Hodgkin disease. We report on a case of a woman with Hodgkin lymphoma treated with ABVD, who developed 4 years later T‐cell NHL with both nodal and extranodal involvement. Brentuximab vedotin could be an effective choice in treating metachronous T‐cell NHL.

## Graphical abstract

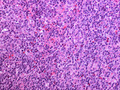

## 1 CASE REPORT

Non‐Hodgkin lymphomas and Hodgkin lymphomas concurrency in the same patient has been noted for several decades.^1^, ^2^, ^3^ All types of NHLs could be found, but the most common association is between diffuse large B‐cell lymphoma (DLBCL) and nodular lymphocyte predominant Hodgkin lymphoma (NLPHL).^4^ Some patients with previous HL can also develop T‐NHL in up to 33% of cases.^1^, ^5^, ^6^, ^7^ We discuss the case of a young woman with T‐NHL following previous classical Hodgkin lymphoma (cHL), its features, clinical presentation, and chosen treatment.

A 34‐year‐old female came to our attention on December 2011 suffering from night sweating and enlarged left side neck lymph nodes. Excisional biopsy was performed, and the patient was diagnosed with cHL nodular‐sclerosis variant type 1, stage IIIB. Lymph node was characterized by a marked capsular fibrous thickening and by an expansion of the paracortex that contains several Reed‐Sternberg cells CD30+, CD15+, with weak nuclear expression of PAX5 and negativity for CD20 and CD3. The percentage of CD68 + macrophages was <5% of cellularity. She started ABVD (adryamicin, bleomycin, vinblastine, dacarbazine) chemotherapy regimen on January 2012 for 6 cycles (last cycle on July 2012), and she obtained complete remission (CR) with a negative CT/PET scan.

Four years later, on November 2015, she started suffering from severe abdominal pain, weight loss, diarrhea, and hypergammaglobulinemia with hypereosinophilia (FIP1L1‐PDGFR fusion gene negative). Intestinal parasites and bacteria all tested negative. Suspecting a relapse of HL, we performed a CT/PET scan that showed positive pulmonary opacities and enlarged lymph nodes in mediastinum and abdomen. A mediastinal lymph node excisional biopsy was performed in association of bronchoalveolar lavage and routine blood tests with microbiological researches, showing that pulmonary uptake was consistent with pulmonary aspergillosis.

**Figure 1:**
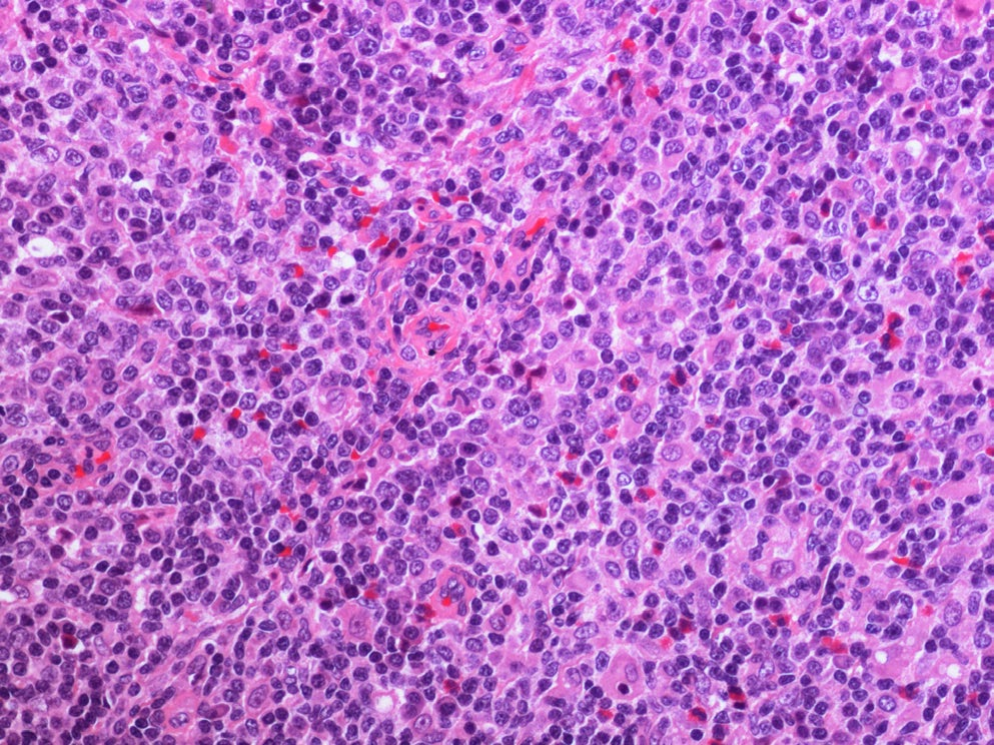
Lymph node biopsy, EE (20×). Polymorphous cells composition, with medium to large atypical cells and an admixture of reactive cells including small lymphocytes and eosinophils

**Figure 2:**
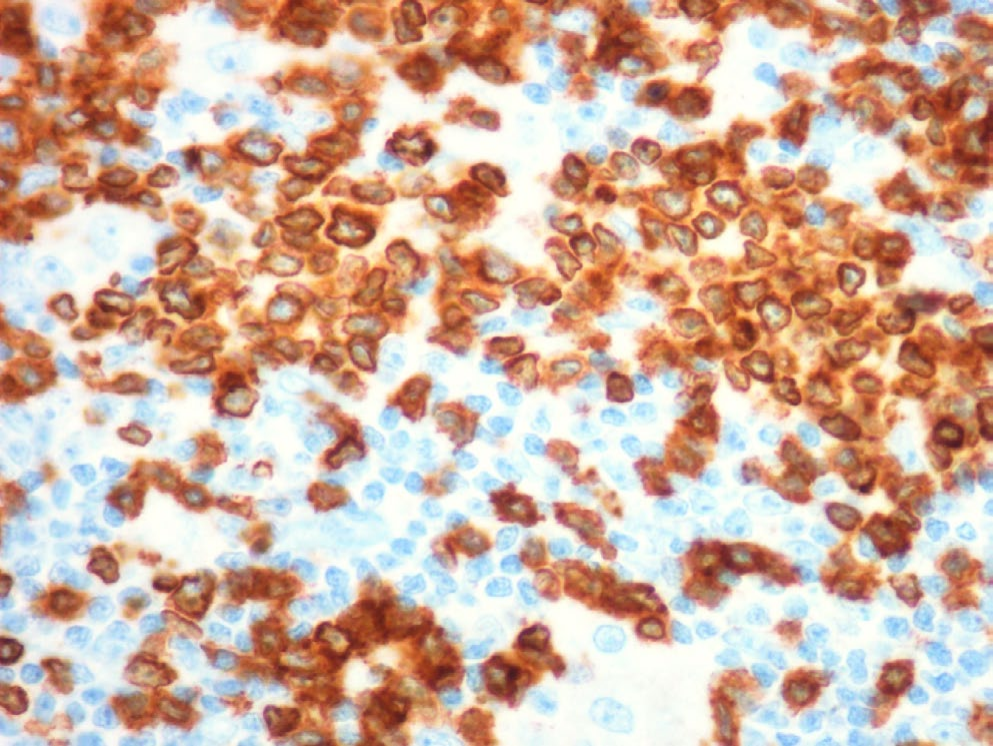
CD3 immunostaining (40×) highlights the small and large cells

**Figure 3:**
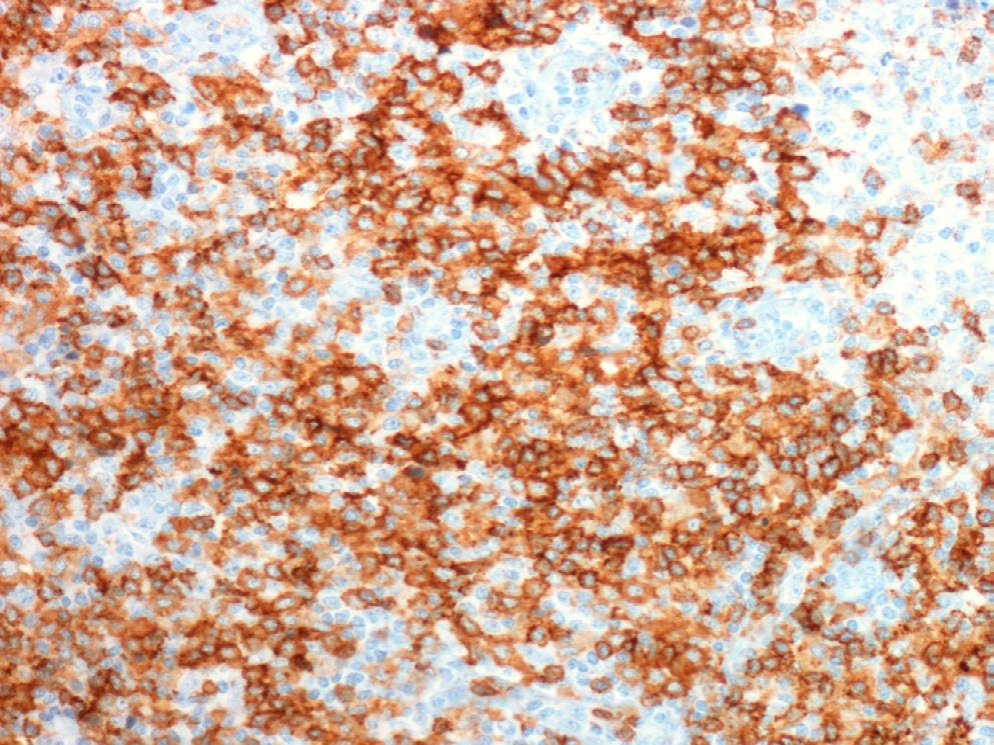
CD4 immunostaining (20×)

To rule out gastrointestinal (GI) involvement, the patient underwent gastric and colorectal endoscopy. Both the results of GI and lymph nodes biopsies were consistent with peripheral T‐cell lymphoma (PTCL), unspecified (NOS), TCR‐betaF1+, CD4+, CD56+/−, and CD3+. Lymph nodes were mainly site of reactive hyperplasia of germinative centers; in the interfollicular area, large atypical, mononuclear, or binucleate cells were observed, with irregular nuclei, sometimes with recesses, dispersed chromatin, and central hyperchromatic nucleolus, associated with a medium‐sized lymphocyte population. Immunohistochemistry revealed the following: CD20−, CD3+, CD30+, CD56+, CD4+, CD8−, CD5+, TCRbetaF1+, ALK−, EMA−, GranzymeB−, PAX5−, TdT−, CD15−, CD21−, OCT2−, BOB1−, CD15−, and BCL6−. In situ hybridization for EBV was negative. Gastric biopsy showed antral gastric mucosa, at the level of the lamina propria, with infiltration consisting of atypical lymphoid cells of medium and large size, with irregular nucleus, evident nucleolus, and eosinophilic cytoplasm. Immunohistochemistry: CD20−, CD3+, CD56+, CD4+, CD8−, CD30+/−, TCRbetaF1+, PAX5−, and GranzymeB−. Histological review was being made with confirmation of PTCL NOS diagnosis. Bone marrow aspirate and biopsy did not reveal pathological infiltration. Both gastric and lymph node biopsies were reviewed by another pathologist who confirmed the diagnosis of PTCL NOS (Figures 1, 2, 3).

**Figure 4:**
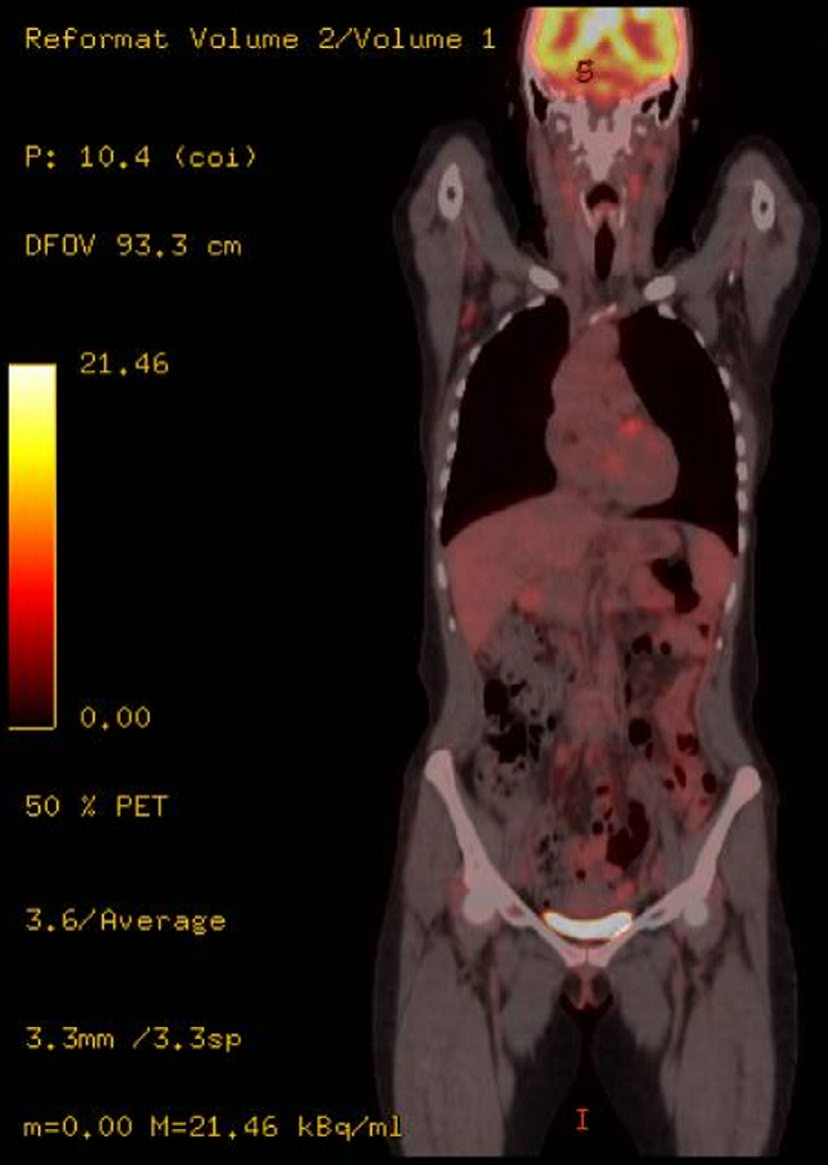
CT/PET scan before BV treatment

**Figure 5:**
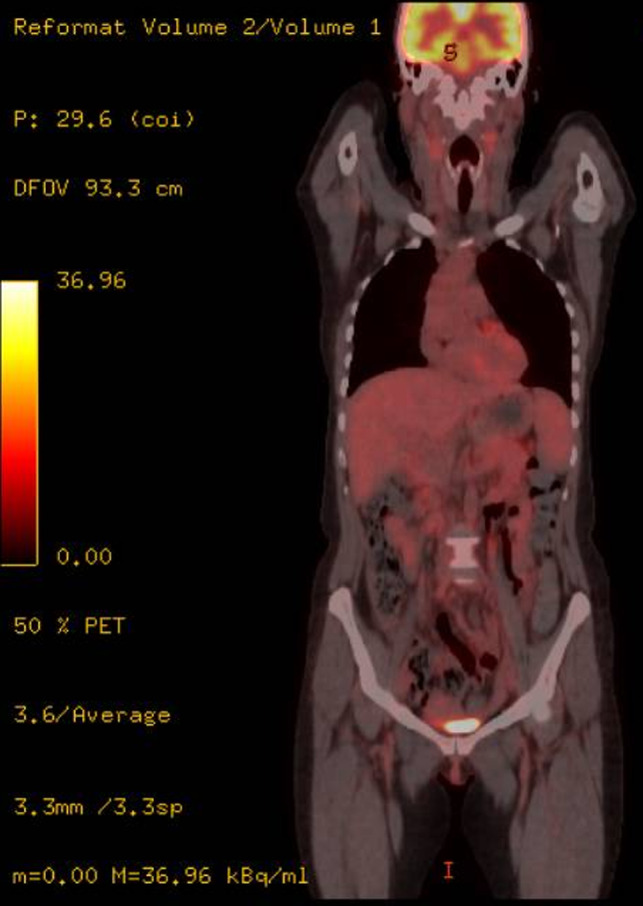
CT/PET scan after BV treatment

On February 2016, the patient started a polychemotherapy regimen^8^ consisting in 1 cycle of CHOP with 3 cycles of IVE (ifosfamide 3 g/m^2^ on day 1‐3; epirubicin 50 mg/m^2^, on day 1; etoposide 200 mg/m^2^, on day 1‐3), alternating with intermediate‐dose MTX (1.5 g/m^2^ on day 1). Final CT/PET scan and GI endoscopies were negative, so she underwent autologous stem cell transplantation (ASCT) on October 2016. CT/PET scan at day + 100 from ASCT confirmed CR with negative endoscopies and bone marrow samples. Due to high risk of relapse, an allogeneic stem cell transplantation was planned to consolidate response. In April 2017, CT/PET scan (Figure 4) before allogeneic stem cell transplantation showed the reappearance of enlarged lymph nodes spread to both sides of the diaphragm with also a nodular lesion of the spleen, so she underwent lymph node biopsy that confirmed relapse of PTCL NOS with the same immunohistochemistry and morphologic features of the previous ones (GI biopsies were negative). Since the lymphomatous infiltration was CD30+, the patient started treatment with brentuximab vedotin (BV) from May to June 2017^9^ with no particular toxicity (except of pancreatic laboratory tests increase which resolved delaying the third course of BV). CT/PET scan after BV treatment in July 2017 showed complete remission (Figure 5) on PTCL NOS, so the patient underwent allogeneic stem cell transplantation from unrelated donor in August 2017. She developed cutaneous and intestinal acute graft‐vs‐host disease (stage IV), successfully treated with corticosteroids and infliximab. The patient died for pneumonia and sepsis in January 2018.

## 2 DISCUSSION

Cases reported in literature regarding synchronous and metachronous HL and NHL are not very frequent. However, there are enough data to speculate on their pathogenesis, treatment, and future directions. HL has been known to cause immunodeficiency^10^ and immunological disregulation due to its pathological substrate with Reed‐Sternberg (RS) cells accompanied by several inflammatory T and B nonpathological cells.^11^ PTCL lymphomas coming several years after HL chemotherapy are an unlikely evolution of the same pathological HL cells. They are CD3+ in only 10%‐20% of cases, and in molecular studies, they have clonal immunoglobulin rearrangement, indicating only aberrant expression of T cells antigen but not their T lineage.^12^, ^13^, ^14^ Arguably, the continuous stimulation of EBV virus (even if it does not integrate in T cell's DNA) causes the mysregulation and abnormal proliferation of oligoclonal T cells, resulting in a clonal expansion of those previously genetically damaged by HL chemotherapy. It seems likely, therefore, that T‐NHL with previous chemotherapy for HL could derive from T cells, with genetic alterations and damages due to chemotherapy agents (therapy related), facilitated to emerge and evolve in clonal cells by their interaction with EBV virus. Further studies are necessary to understand the pathogenesis and the most appropriate treatment of this metachronous T‐NHL.

## CONFLICT OF INTEREST

The authors declare that there is no conflict of interest regarding the publication of this paper.

## AUTHORSHIP

All authors contributed equally to this manuscript.
